# Molecular characterization of HTLV-1 and HTLV-2 and routes of virus transmission in HIV-infected patients from the southeastern and southern Brazil

**DOI:** 10.1186/1742-4690-11-S1-P47

**Published:** 2014-01-07

**Authors:** Mariana C Magri, Luis FM Brígido, Helena K Morimoto, Adele Caterino-de-Araujo

**Affiliations:** 1 Laboratório de Investigações Médicas em Hepatologia por Vírus (LIM-47), Faculdade de Medicina, Universidade de São Paulo, Brazil; 2 Laboratório de Retrovirus, Centro de Virologia, Instituto Adolfo Lutz, Secretaria de Estado da Saúde de São Paulo, Brazil; 3 Departmento de Patologia, Análises Clínicas e Toxicológicas, Universidade Estadual de Londrina, Londrina, PR, Brazil; 4 Centro de Imunologia, Instituto Adolfo Lutz, Secretaria de Estado da Saúde de São Paulo, São Paulo, SP, Brazil

## 

Brazil is considered the country with the largest absolute number of individuals infected with HTLV-1/2, close to 2,500,000, and is also considered epidemic for HIV/Aids with a prevalence rate of 0.3%-0.6%. The routes of such retroviruses transmission could vary according to the region, population and period of study. This study aimed to characterize HTLV-1 and HTLV-2 that circulate in HIV-coinfected patients from the southern (n=34) and southeastern (n=20) Brazil. For analysis, sequencing of LTR, env and tax regions of HTLV-1 and HTLV-2 and bioinformatic tools were employed, and the results obtained analyzed according to geographic regions and risk factors. The results obtined confirmed HTLV-1a subtype, Transcontinental subgroup A as the prevalent, grouping into two Latin America clusters. The most frequent nucleotide substitution V1981I in the env and the Brazilian molecular signature in TaxA were detected. The HTLV-2a subtype, variant 2c (characteristic of the long Tax found in Brazilian strains) was detected in all except one isolate, as well the amino acid change S1909P in the env region. The HTLV-2 clustered according to geographic region and risk factors (sexual and IDU). All sequences are in GenBank. This study confirmed the double entrance of HTLV-1 in Brazil, and the presence of the Amerindian HTLV-2c in HIV-coinfected patients. The lack, in the present study, of the prototypic North American HTLV-2a and HTLV-2b subtypes circulating worldwide suggest the introduction of HTLV-2 before HIV-1 in such vulnerable population of Brazil. Support: MCT/CNPq # 481040/2007-2 and # 303545/2012-7, CAPES, IAL # 33/07 and # 39/07), Brazil.

